# Diagnostic accuracy of ultrasound for dysphagia in neurological disorders including stroke: a systematic review and meta-analysis

**DOI:** 10.3389/fneur.2025.1534173

**Published:** 2025-08-21

**Authors:** Liu Yang, Dongxue Liu, Dai Shuang, Liang Shuang, Yujiao Wang, Lan Gao

**Affiliations:** Department of Neurology, The First Hospital of Jilin University, Changchun, Jilin, China

**Keywords:** ultrasound, dysphagia, severe dysphagia cases, swallowing evaluation, stroke

## Abstract

**Objective:**

To investigate the diagnostic accuracy of ultrasonography in detecting dysphagia and to compare it with other diagnostic methods.

**Methods:**

This is a systematic review and meta-analysis of observational studies. The literature was searched in multiple databases, including the Cochrane Central Controlled Trials Registry (a global database of controlled trials); MEDLINE, EMBASE, and Web of Science (biomedical, pharmacological, and multidisciplinary citation databases, respectively); CINAHL (focusing on nursing and allied health research); and Chinese databases including Wanfang Data, CNKI, and VIP (covering academic, scholarly, and scientific-technical literature). Only articles published in the English and Chinese languages were included. Studies were eligible if they compared the accuracy of ultrasound testing with that of other diagnostic methods in dysphagia patients. The Quality Assessment of Diagnostic Accuracy Studies (QUADAS-2) criteria (a tool for evaluating bias risk in diagnostic accuracy studies) were used to assess the risk of bias following standard procedures.

**Results:**

We included eight studies involving a total of 538 patients with dysphagia: seven trials for post-stroke dysphagia and one trial for dysphagia in children with cerebral palsy. The combined results showed that the sensitivity and specificity of ultrasound were 0.81 (95% CI 0.73–0.87) and 0.86 (95% CI 0.76–0.93), suggesting that the diagnostic performance of ultrasound is reliable for detecting dysphagia in patients.

## 1 Introduction

Dysphagia, also known as disordered swallowing, refers to an abnormality that occurs when food travels from the mouth to the stomach (1). This can be caused by a variety of factors, such as neuromuscular diseases—particularly stroke and cerebral palsy—trauma, surgery, or aging. The nervous system plays a pivotal role in this process: swallowing relies on precise regulation of oral, pharyngeal, and esophageal muscles by central neural circuits (e.g., cortical swallowing centers and brainstem nuclei), with disruptions with neurological disorders often leading to dysfunction (2), For instance, stroke-induced dysphagia arises from acute damage to neural pathways, causing muscle paresis or reflex impairment, while cerebral palsy—rooted in developmental brain injury—manifests as spasticity or ataxia in swallowing muscles, disrupting food manipulation and laryngeal closure. Swallowing disorders not only affect an individual’s nutritional intake and quality of life, but can also lead to serious complications such as aspiration, aspiration pneumonia, and even malnutrition and dehydration. Therefore, early recognition and accurate diagnosis of swallowing disorders—especially those linked to neurological etiologies—are essential for timely intervention and treatment.

Ultrasound is one tool that can be used alongside other assessments, e.g., bedside swallowing assessments. In clinical scenarios, especially when evaluating patients with potential swallowing disorders, combining ultrasound with bedside swallowing assessments proves highly beneficial. The bedside swallowing assessments can offer on-the-spot, practical observations of a patient’s swallowing actions during care. Meanwhile, ultrasound provides detailed, real-time visual data regarding the physiological mechanisms of swallowing. This integration of the two approaches amplifies diagnostic accuracy and paves the way for more precisely targeted treatment plans (3).

Although ultrasonography shows promise for diagnosing swallowing disorders, its accuracy requires validation through large-scale clinical studies. Current gold standard methods like VFSS (Videofluoroscopic Swallowing Study) and FEES (Fiberoptic Endoscopic Evaluation of Swallowing) are effective but costly, complex, or invasive (4). Thus, the role of ultrasound as an alternative or adjunctive tool for dysphagia diagnosis remains unclear and warrants systematic review.

The purpose of this systematic review and meta-analysis was to synthesize existing clinical studies to evaluate the accuracy of ultrasonography in diagnosing dysphagia. By quantitatively analyzing the sensitivity and specificity of ultrasound and comparing it with the existing diagnostic methods, this study will provide the scientific basis for the application of ultrasound in the diagnosis of swallowing disorders and point out the direction for future clinical practice and research.

## 2 Methods

The methodology follows the approaches outlined in the “Cochrane Handbook for Systematic Reviews of Interventions (5)“and the “Cochrane Handbook for Diagnostic Test Accuracy,” (6) with registration number: CRD42024550532.

### 2.1 Inclusion and discharge standards

We considered controlled diagnostic test accuracy studies, such as prospective cohort, cross - sectional, and case - control studies, which compare the diagnostic accuracy of ultrasound with other diagnostic methods for dysphagia. The reference standards for these comparisons include videofluoroscopic swallowing study (VFSS), Functional Oral Intake Scale (FOIS), Kotani’s Drinking Test, swallowing contrast examination, and the results of the Dysphagia Disorder Survey Questionnaire (DDS)/Oral Motor Assessment, as these examinations are used as the gold standard for assessing dysphagia in clinical settings (7).

All patients with dysphagia, including children, who underwent ultrasound examination were included in the scope of our study. We included all studies on ultrasound examination for dysphagia that met the above - mentioned inclusion criteria. The target condition of our research is all patients requiring ultrasound diagnosis for dysphagia.

We excluded uncontrolled reports (case series, case reports), studies on ultrasound - guided dysphagia treatment, and review studies. Additionally, studies in which the diagnostic accuracy of ultrasound, such as specificity or sensitivity, was not recorded or could not be calculated were also excluded. This measure was taken to ensure that our research remained focused and precise, thereby simplifying the analysis and improving reliability.

### 2.2 Search methods for identification of studies

#### 2.2.1 Electronic search

After consulting scholars well-versed in systematic reviews for their suggestions, we formulated a search strategy specifically tailored for retrieving relevant literature. Given the distinct search features of various databases, we meticulously referred to and adhered to the guidelines outlined in the Cochrane Handbook of Systematic Reviews of Interventions to design diverse search approaches.

Our search terms in both Chinese and English encompassed “dysphagia,” “swallowing,” “assessment diagnosis,” “ultrasound,” “ultrasonography,” and “ultrasonics.” The Chinese search terms were carefully selected in line with the terminologies commonly used in China.

It should be noted that our search was restricted to articles published in either Chinese or English. We imposed no limitations on the publication date, aiming to conduct an exhaustive search across each of the following databases: Cochrane Central Register of Controlled Trials, MEDLINE, EMBASE, CINAHL, and Web of Science. We also searched three Chinese databases: Wanfang Data, CNKI, and VIP, from their inception to September 2024, with as broad a search range as possible.

#### 2.2.2 Search of other resources

We screened the reference lists of relevant trials to identify any further potential papers worth summarizing.

### 2.3 Data collection and analysis

#### 2.3.1 Study selection

After removing duplicates, the search results were exported to Endnote software (n = 8,679). Two authors (YL and LDX) independently screened the titles and abstracts of articles identified by the search strategy. YL and LDX retrieved the full text of potentially eligible articles and independently assessed their relevance. Disagreements about eligibility were resolved by consensus throughout the selection process.

#### 2.3.2 Data extraction and management

Two reviewers (YL and LDX) independently extracted data on study characteristics, patient demographics, sample size, testing methods, methodological quality, sensitivity, and specificity. They then constructed a 2×2 contingency table with the extracted data.

#### 2.3.3 Assessment of method quality

We used the QUADAS-2 tool to assess the risk of bias in the included studies, as outlined by Whiting et al. (8) and recommended by the Cochrane Diagnostic Test Accuracy Working Group (9). The studies were described with judgments of “low,” “high,” or “unclear” for each item in the tool, and an overall rating for each domain was given. We considered studies classified as “low risk of bias” and “low concern” in all domains to have high methodological quality. The assessment of methodological quality was conducted independently by two reviewers, and the final decision was made by consensus.

#### 2.3.4 Statistical analysis and data synthesis

Data synthesis was conducted using the methods recommended by the Cochrane Collaboration’s Diagnostic Test Accuracy Working Group (6). Forest plots were used to display the number of true positives (TP), true negatives (TN), false positives (FP), and false negatives (FN) of all included studies, as well as sensitivity and specificity with their 95% confidence intervals (CI) (10). We also used a summary receiver operating characteristic (SROC) plot to display the results of individual studies in the ROC space, with each study plotted as a single sensitivity-specificity point. In the meta-analysis, we used a bivariate random-effects model to determine the pooled estimates of sensitivity and specificity with 95% confidence intervals and prediction regions. The clinical application of ultrasound examination was evaluated using the likelihood ratio, and the post-test probability (based on Bayes’ theorem) was calculated using Fagan’s nomogram with Review Manager 5 software and Stata 17 for statistical analysis (11).

### 2.4 Investigation of heterogeneity

Heterogeneity was examined through visual inspection of sensitivity and specificity in the forest plot and I^2^ statistical results. We explored potential sources of heterogeneity through subgroup analysis and meta-regression (including sample size, different reference indicators, and study types). Heterogeneity is mainly caused by threshold effects and/or non-threshold effects. If there is a strong positive correlation between the natural logarithm of sensitivity and (1-specificity) with a *p*-value <0.05, it indicates the presence of a threshold effect, and only the AUC value is calculated; otherwise, if there is no threshold effect, further testing of heterogeneity caused by non-threshold effects is performed. Non-threshold effects were tested using the chi-square test or the Cochrane-Q test, and the size of heterogeneity among studies was assessed using the I^2^ value, where I^2^ < 25% indicates low heterogeneity, 25% ≤ I^2^ < 50% indicates low to moderate heterogeneity, 50% ≤ I^2^ < 75% indicates moderate heterogeneity, and I^2^ > 75% indicates high heterogeneity. If *p* > 0.1 and I^2^ < 50%, it indicates no heterogeneity among multiple studies, and a fixed-effects model is used for the combination of effect sizes; otherwise, a random-effects model is used for combined analysis, and meta-regression and sensitivity analysis are performed to explore the sources of heterogeneity.

### 2.5 Sensitivity analysis

We performed a sensitivity analysis using a “leave-one-out” procedure to check the degree of change in the results due to data variation; that is, by removing a single study from the meta-analysis each time to reflect the impact of a single dataset on the pooled results.

### 2.6 Assessment of reporting bias

The symmetry of the funnel plot can be used to measure whether there is publication bias in the included original studies. We used Stata 15.0 software to draw Deek’s funnel plots (Deek’s funnel plot asymmetry test) for CEUS and HR-MRI. If the funnel plot shows a result of *p* ≥ 0.05, it indicates no publication bias; if the funnel plot shows a result of *p* < 0.05, it indicates the presence of publication bias.

## 3 Results

### 3.1 Study description

**Figure 1:**
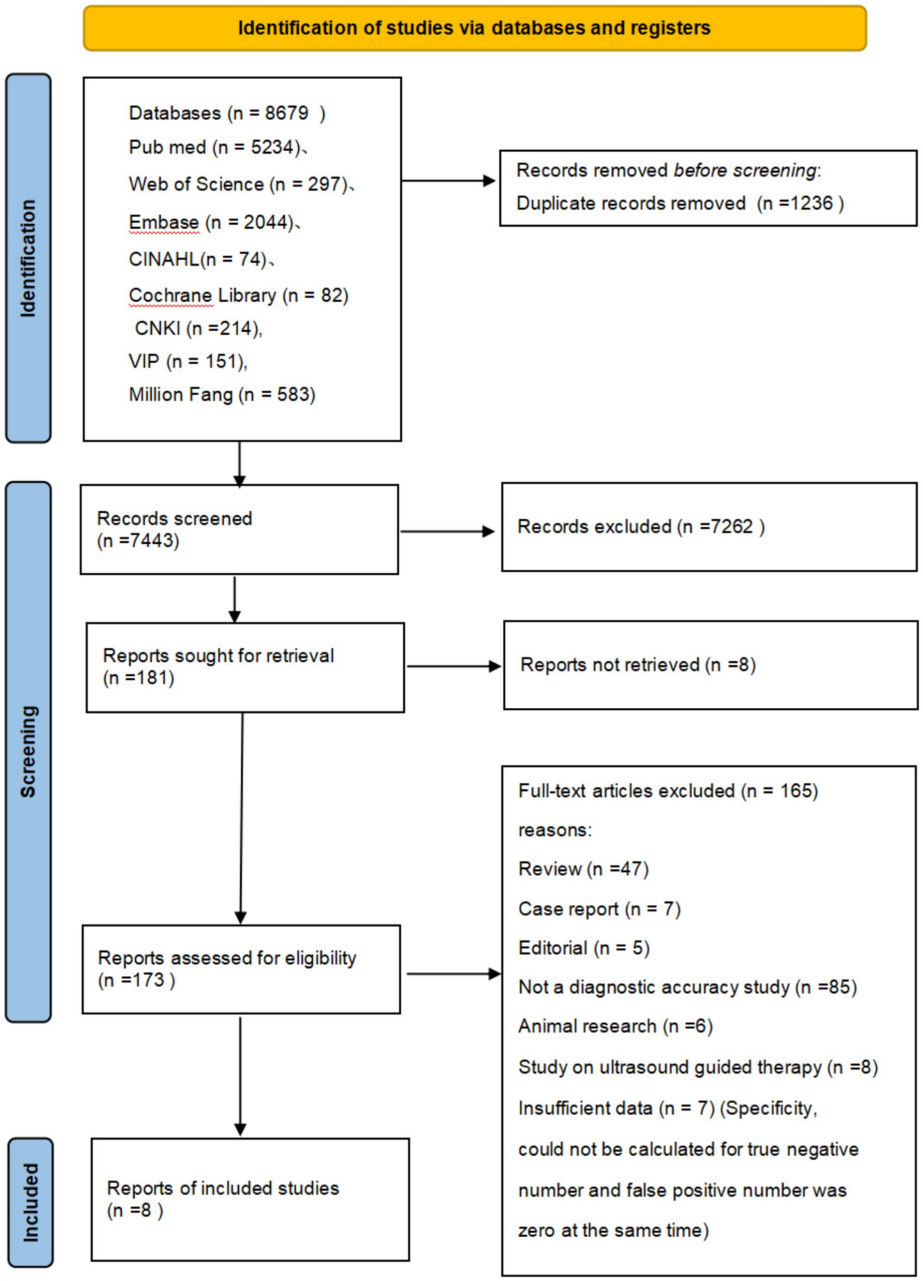
PRISMA flow diagram of the study selection process.

We retrieved 8,679 articles through electronic searches from the following databases (English *n* = 7,731, Chinese *n* = 948): Cochrane Central Register of Controlled Trials (Central) (*n* = 82), MEDLINE (via PubMed) (*n* = 5,234), EMBASE (OvidSP) (*n* = 2044), Web of Science (*n* = 297), CINAHL (via EBSCO) (*n* = 74), Wanfang Data (*n* = 583), CNKI (*n* = 214), and VIP (*n* = 151). No articles were identified through scanning the reference lists of the identified studies. After excluding duplicates, there were 7,262 citations left. Figure 1 shows the flow of citations in the selection process.

We retrieved 181 full-text articles for assessment. The main reasons for excluding full-text articles were study design issues, non-study nature, non-diagnostic accuracy studies, animal studies, ultrasound-guided catheterization studies, or review articles. Additionally, eight studies could not be retrieved and were excluded, and another seven potential studies were excluded because specificity could not be calculated. Finally, eight studies (English *n* = 3, Chinese *n* = 5) were included in this review.

All included studies were published after 2009, two were clinical trials (12, 13), and the remaining six were cross-sectional studies (14–19). These studies recorded the diagnostic accuracy of ultrasound compared to other diagnostic methods as the reference standard (e.g., specificity or sensitivity). A total of 538 patients were enrolled in two different countries.

A total of 8 studies were included, 7 of which were conducted in China (12–18), and 1 in Japan (19). Among them, 7 studies involved stroke patients (12, 14–19), and 1 study involved children with cerebral palsy (13). The parts and indicators used for ultrasound detection in the included studies varied (13, 15). Two studies used hyoid bone displacement amplitude for diagnosis, two studies used hyolaryngeal movement ratio, one study used hyoid-thyroid cartilage distance shortening rate (ASR) (17), one study used M-mode swallowing curve ascending phase motion time, another study referred to the speed of the tongue’s downward (Vd) and upward (Vu) movement during the swallowing phase (12), and the distance from the tail depression to the skull elevation position (D) (19).

Five studies adopted the gold standard videofluoroscopic swallowing study (VFSS) (14, 16–19), one study used the Dysphagia Disorder Survey Questionnaire (DDS) and oral motor assessment results to diagnose dysphagia in children with cerebral palsy (13), one study used the Kotani Drinking Test (12), and another study used the Functional Oral Intake Scale (FOIS) (15). Seven studies determined the optimal cutoff value based on the ROC curve (12, 13, 15–19) (Table 1).

**Table 1:** Summary of included studies (*n* = 8).

(Author year)	Country	Sample size experimental group/control group	Disease	Diagnostic index	Reference index	Research type
2017 Xiong Chunhua (13)	China	7/20	Children with cerebral palsy	Hyoid displacement amplitude	Dysphagia scale	Clinical trial
2023 Deng Miaomiao (18)	China	43/43	Stroke	Hyo-laryngeal movement (HL) motion ratio	Videofluoroscopic Swallow Study (VFSS)	Cross-sectional study
2022 Huang Gelang (17)	China	42/40	Stroke	Hypothyroid distance shortening rate (ASR)	Videofluoroscopic Swallow Study (VFSS)	Cross-sectional study
2013 Li Junlai (12)	China	20/91	Stroke	Movement time during the ascending phase of the M hyper swallow curve	Kotani’s Drinking Test	Clinical trial
2021 Takako Matsuo (16)	Japan	18/18	Stroke	Hyo-laryngeal movement (HL) motion ratio	Videofluoroscopic Swallow Study (VFSS)	Cross-sectional study
2011 Yasuhiro Tomii (19)	Japan	24/76	Stroke	The speed with which the tongue moves down (Vd) and up (Vu) during swallowing, and the distance from the tail depression to the elevated skull (D).	Videofluoroscopic Swallow Study (VFSS)	Cross-sectional study
2012 Xiao Mingyan (15)	China	30/40	Stroke	Hyoid displacement amplitude	Functional Oral Feeding Scale (FOIS)	Cross-sectional study
2009 Su Yiji (14)	China	9/17	Stroke	Lingual indentation	Videofluoroscopic Swallow Study (VFSS)	Case–control

### 3.2 Methodological quality of included research

A QUADAS-2 evaluation was carried out to assess the quality of the eight included studies, which revealed a complex picture of risk assessment across multiple domains.

There were significant defects in all the 8 studies. None of these studies managed to avoid the design of case–control studies, and they failed to include patients who were “difficult to diagnose.” Due to the lack of comprehensive patient selection, there is a high risk of bias in the case selection process in all cases.

Five studies did not indicate whether diagnostic indicators were interpreted without knowing the gold standard results (12, 13, 15–17). Seven studies did not use the pre-set threshold (12, 13, 15–19) but set the optimal threshold based on ROC results, which led to different degrees of risk in the included literature in the field of diagnostic tests.

**Figure 2:**
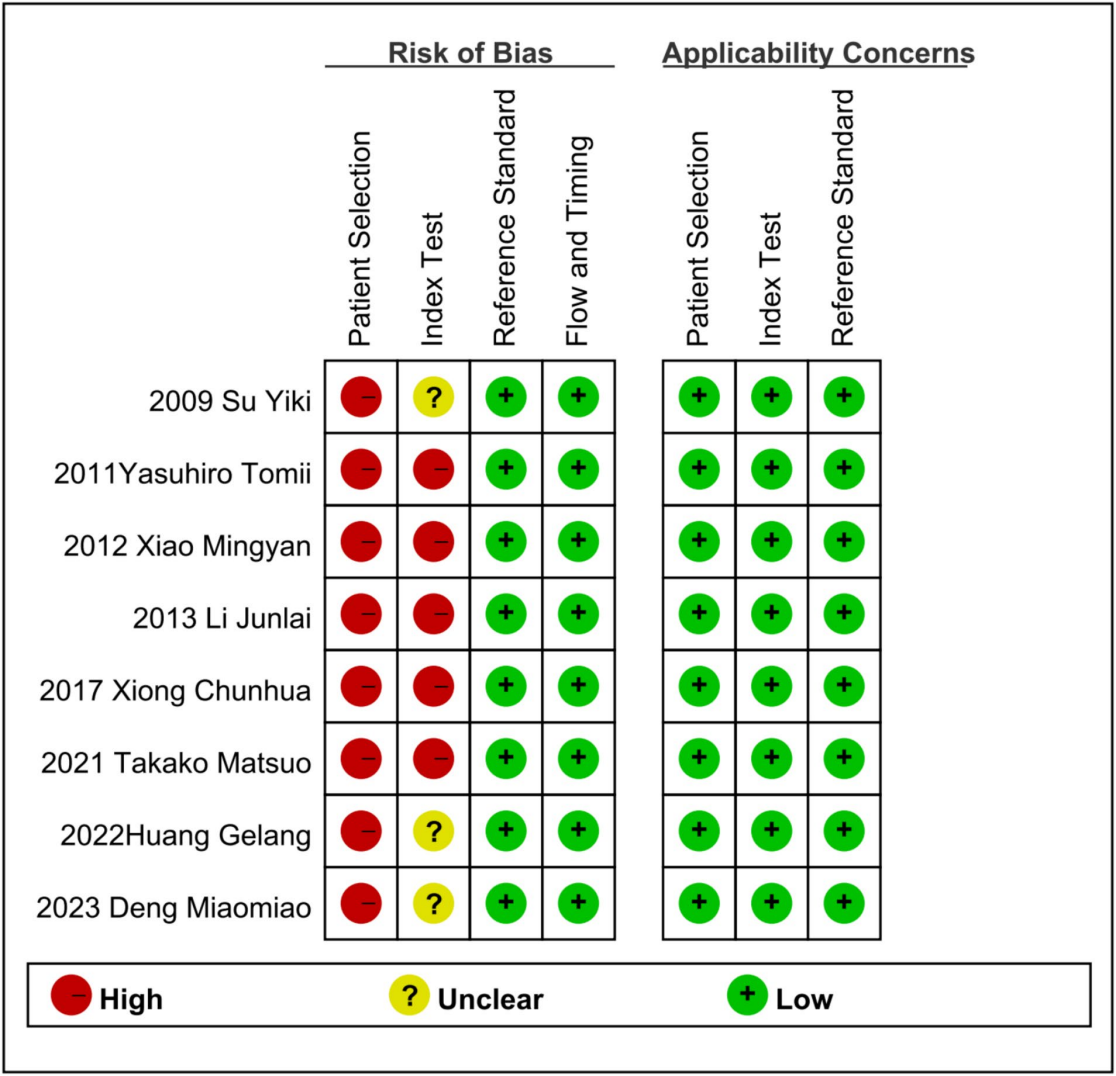
Risk of bias and applicability concerns summary: review authors’ judgments about each domain for each included study.

**Figure 3:**
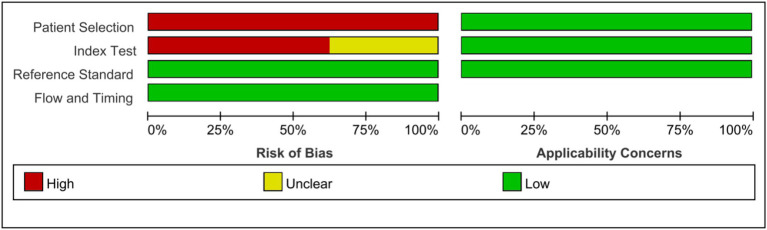
Risk of bias and applicability concerns graph: review authors’ judgments about each domain presented as percentages across included studies.

As shown in the QUADAS-2 results (Figures 2, 3). No questions were raised about applicability, patient selection bias, or interpretation of reference trial results.

### 3.3 Estimation of diagnostic accuracy of dysphagia by ultrasonography

**Figure 4:**
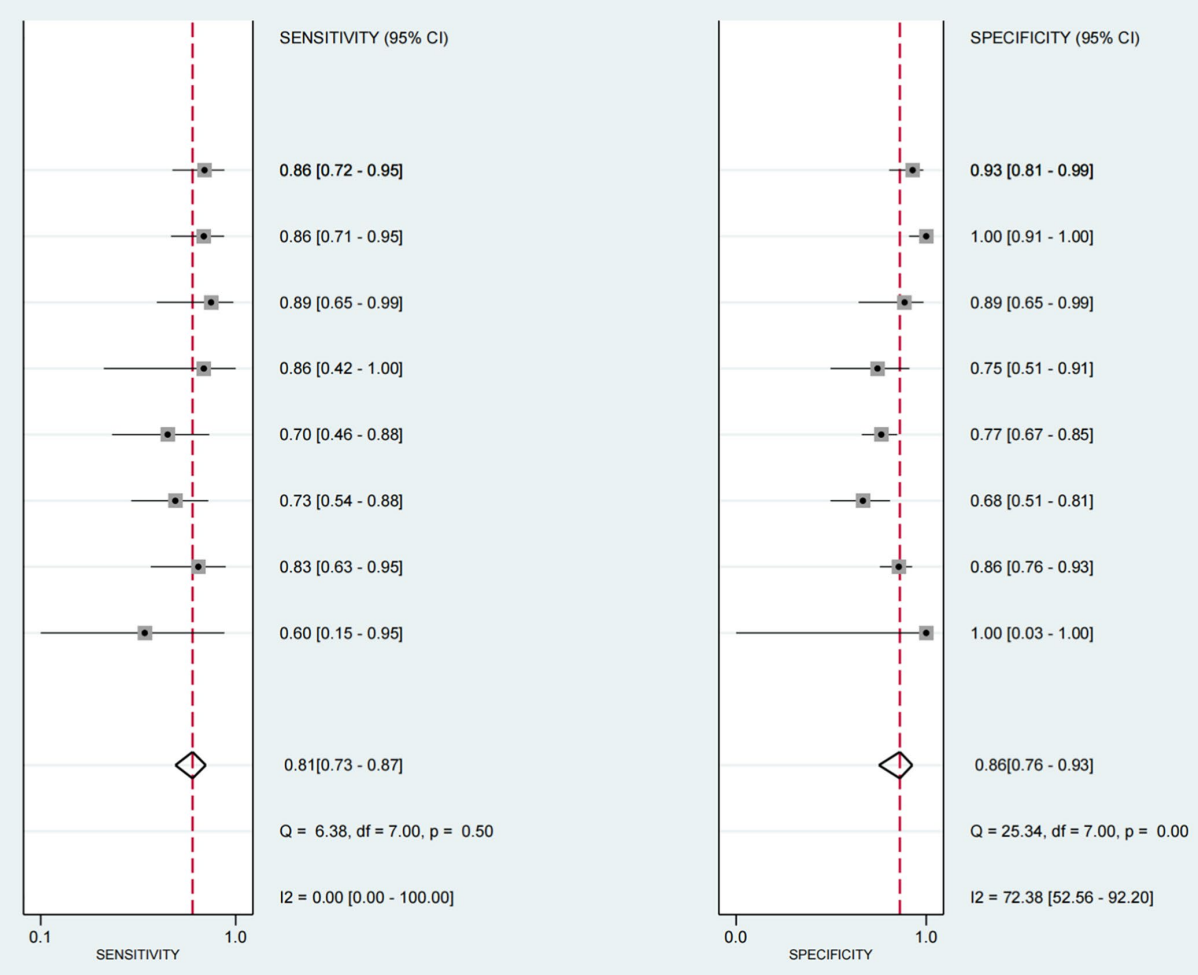
Forest map of sensitivity and specificity of ultrasound - detected swallowing dysfunction (with other criteria as reference). This figure is a forest map presenting the sensitivity and specificity of ultrasound detection of swallowing dysfunction in patients, with other criteria serving as a reference. Values between brackets represent the 95% confidence intervals (CI) for sensitivity and specificity. The gray box in the graph indicates the estimated sensitivity and specificity of the study, and the black horizontal line represents its 95% CI.

**Figure 5:**
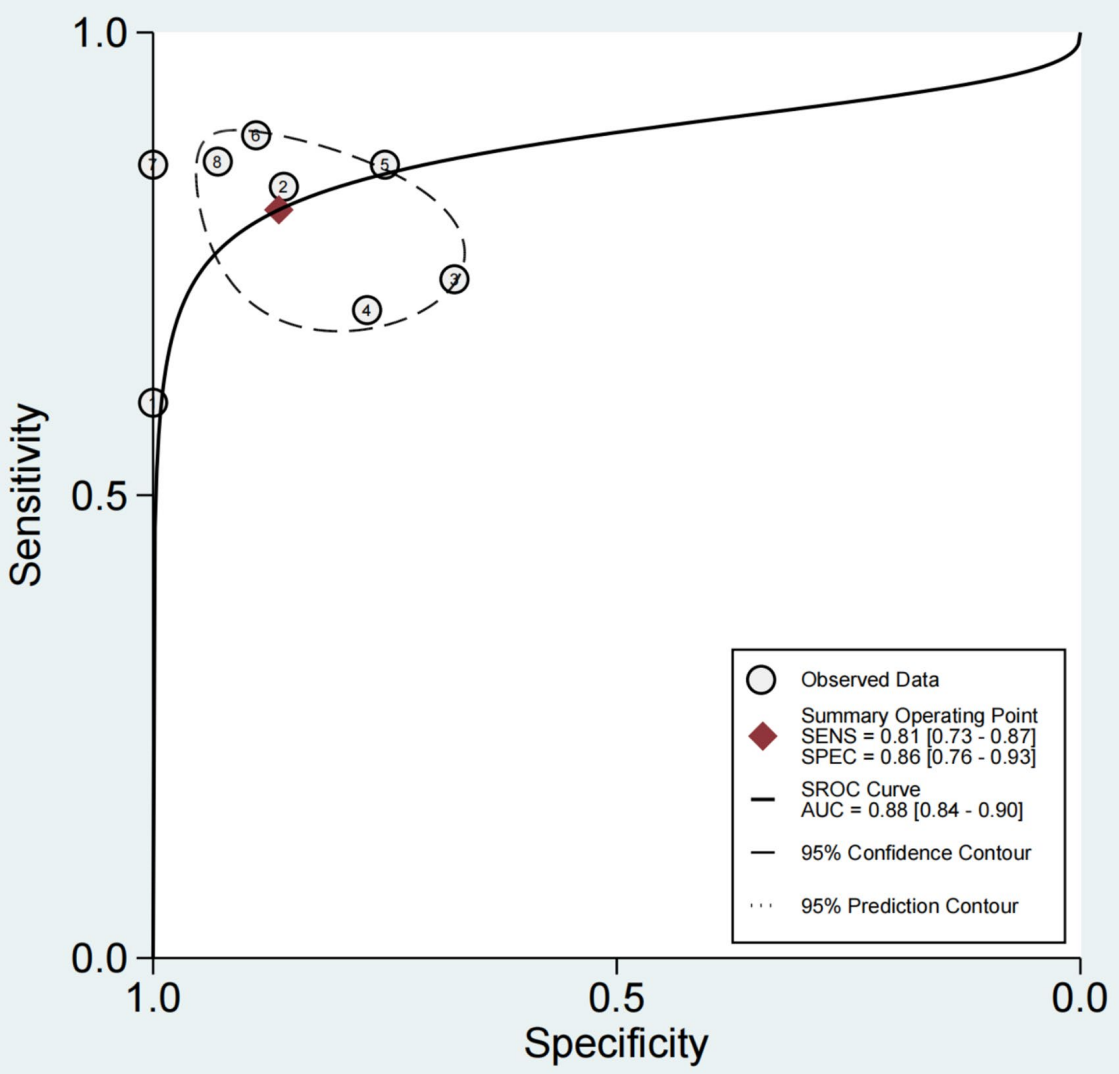
SROC curve with 95% CI and prediction area for 8 studies in ultrasonic diagnosis of swallowing dysfunction. SROC curve with summary point 95% confidence interval (CI) and prediction area for 8 studies in ultrasonic diagnosis of swallowing dysfunction. The full circle corresponds to a pooled estimate of sensitivity and specificity and represents the 95% confidence region (dashed line) and 95% prediction region (dashed line).

The sensitivity and specificity of each study are shown in Figure 4. The SROC curve with the 95% confidence interval (CI) of the summary point and the prediction region are shown in Figure 5. The results included a sensitivity of 0.81 (95%CI 0.73–0.87) and a specificity of 0.86 (95%CI 0.76–0.93). The area under the curve (AUC) was 0.88(95%CI 0.84–0.90), with no heterogeneity among sensitivities and moderate heterogeneity among specificities.

**Figure 6:**
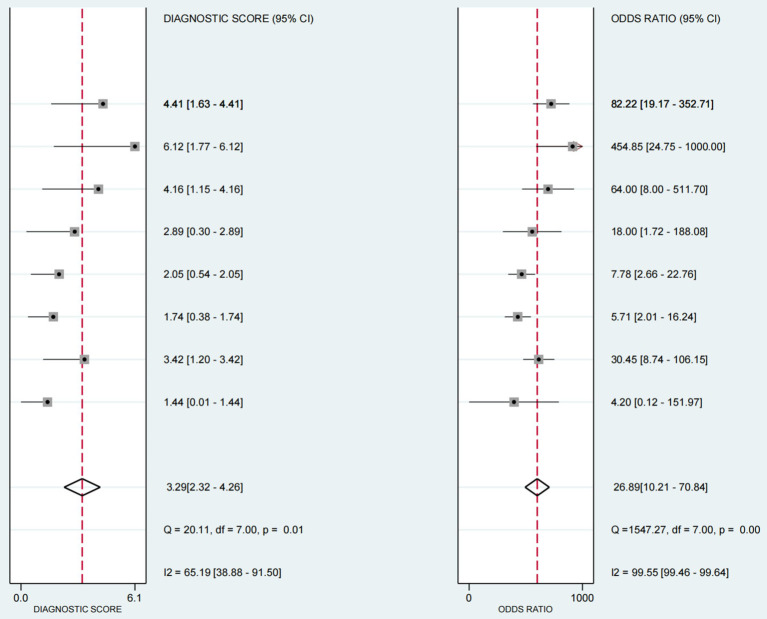
Forest plot of diagnostic scores and odds ratios in ultrasound-based dysphagia assessment (with 95% CI). Diagnostic scores and ratios of each individual study in the assessment of dysphagia by ultrasound. The diagnostic score and odds ratio, each of which provides a 95% Confidence Interval (CI), as well as the results of several statistical tests, such as the Q statistic and the I^2^ statistic.

**Figure 7:**
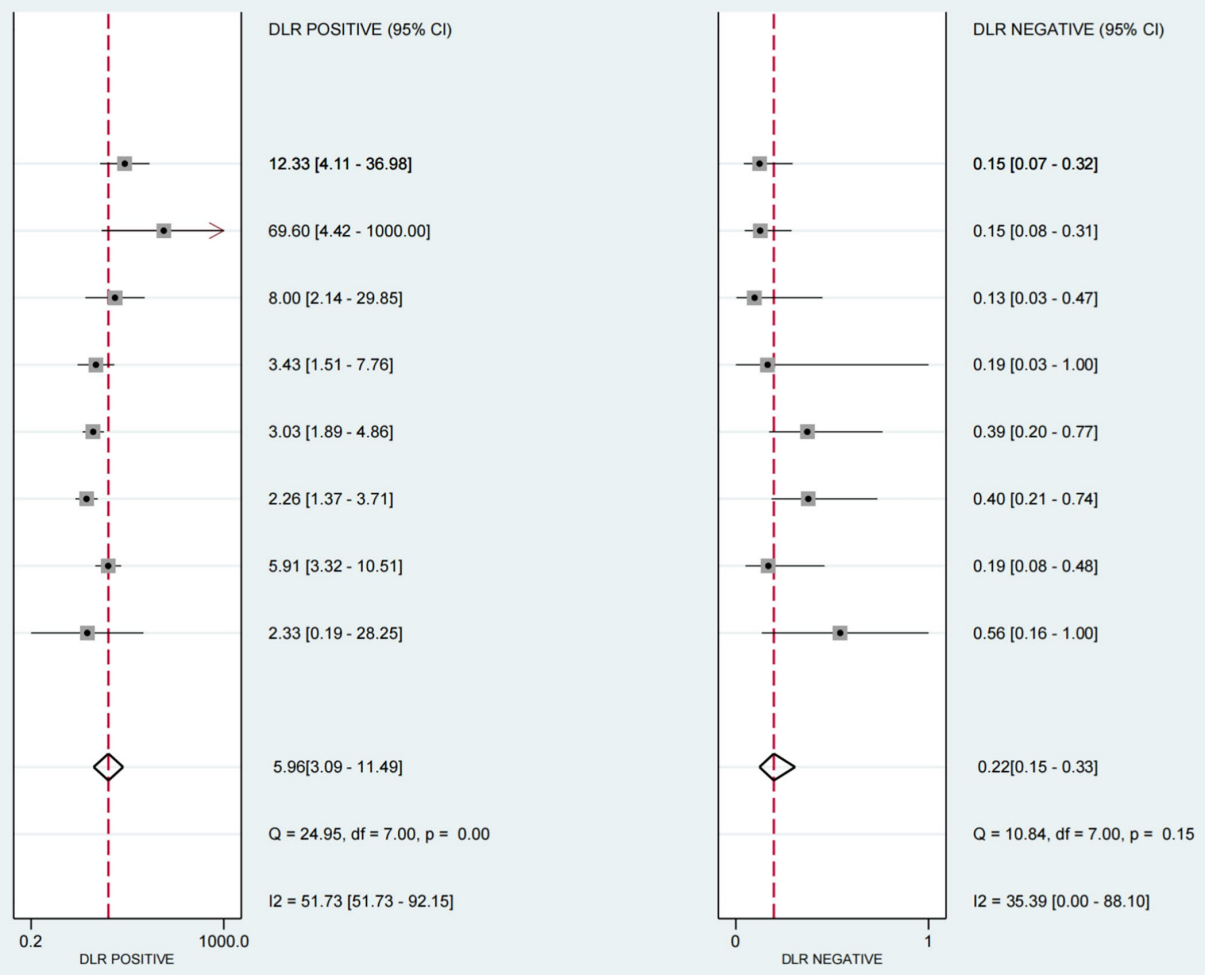
Likelihood of individual studies in ultrasound-based dysphagia diagnosis.

The positive likelihood ratio (PLR) was 5.96 (95%CI 3.09–11.9), the diagnostic odds ratio (DOR) was 26.89(95%CI 10.21–70.84) (see Figure 6), and the negative likelihood ratio (NLR) was 0.22 (95%CI 0.15–0.33) (see Figure 7).

**Figure 8:**
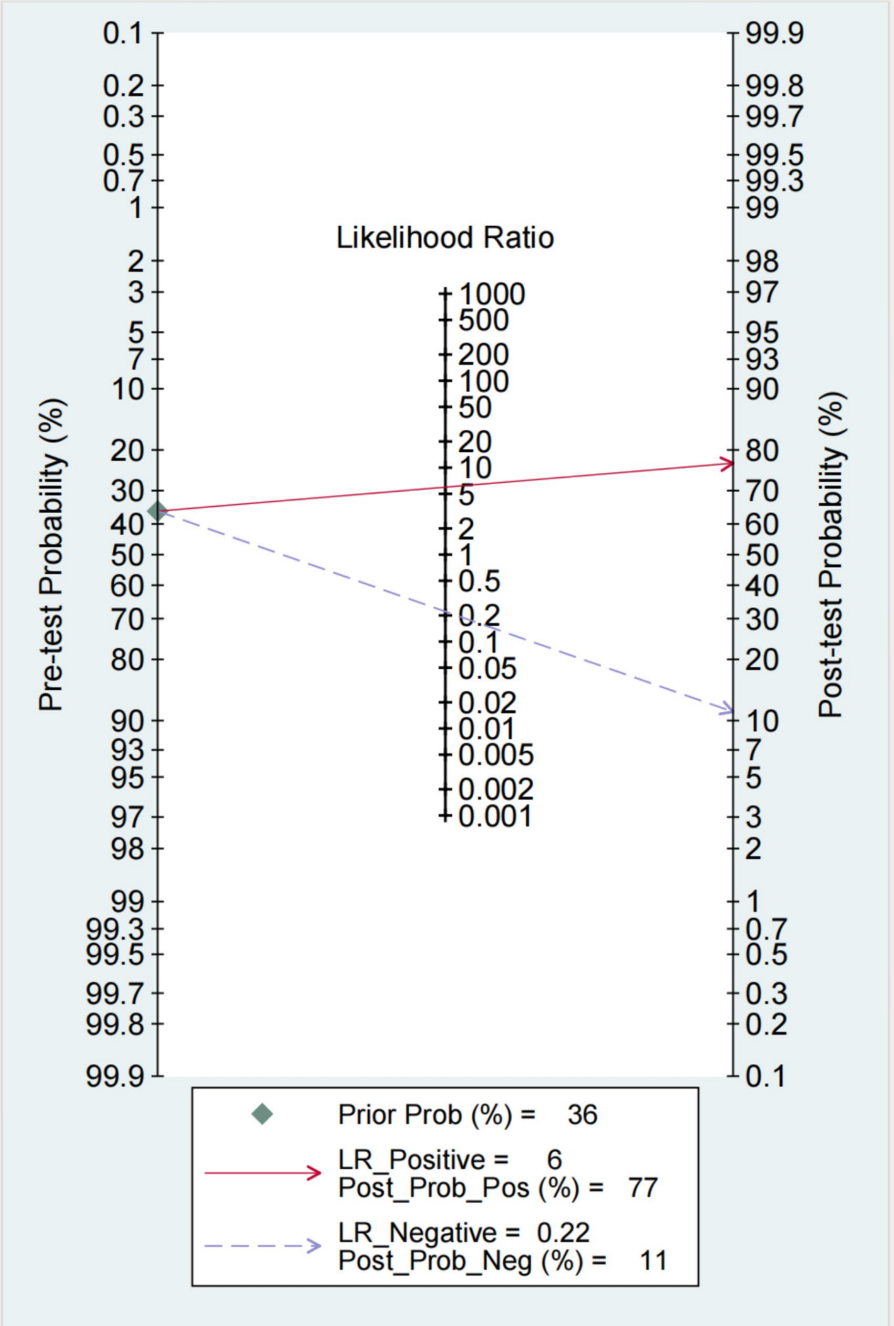
Fagan plot: estimation of the impact of ultrasound results on the probability of swallowing disorder. The Fagan plot estimates the extent to which the ultrasound results alter the probability of swallowing disorder, given the predicted probability.

**Figure 9:**
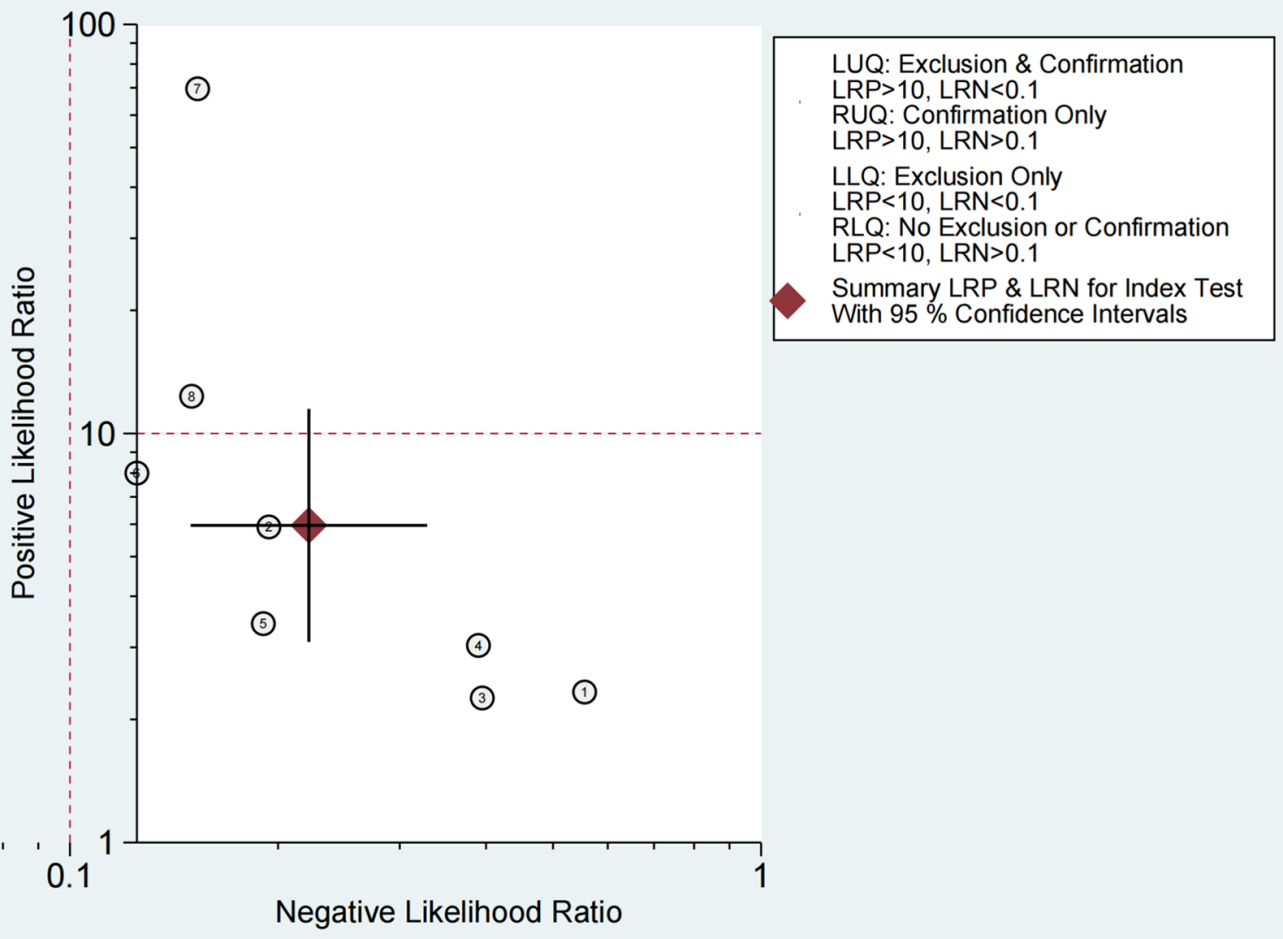
Likelihood ratio scatter plot for ultrasound-based dysphagia diagnosis (with quadrant interpretation).

The Fagan chart (Figure 8) shows that ultrasound may be clinically informative as it increases the probability of previously being classified as N + from 36% for positive (the average prevalence of N + cases) to 100% while reducing the same probability to 11% for negative. As can be seen from the likelihood ratio (LR) scatter plot (Figure 9), the aggregate points of positive and negative LR are located in the lower right quadrant, indicating that ultrasound may not be very helpful in diagnosing dysphagia test results.

Figure 9 shows the ability to diagnose dysphagia by ultrasound. The likelihood ratio (LR) scatter plot for the information quadrant is defined based on the expected thresholds (positive LR > 10, negative LR < 0.1): LUQ (Left Upper Quadrant): Exclusion and validation areas. If a test has LR+ > 10 and LR- < 0.1, then this test result strongly supports that the patient has a disease and can be used to diagnose and rule out the disease. RUQ (Right Upper Quadrant): Confirm only the region. If LR+ > 10 and LR- ≥ 0.1, the test results support that the patient has disease, but are not sufficient to rule out disease. LLQ (Left Lower Quadrant): Only regions are excluded. If LR+ < 10 and LR- < 0.1, the test results do not support the patient’s disease and can be used to rule out disease. RLQ (Right Lower Quadrant): Regions are neither excluded nor confirmed. If LR+ < 10 and LR- ≥ 0.1, the test results are not very helpful for diagnosis,

### 3.4 Heterogeneity survey

As shown in Figure 4, the forest map shows the sensitivity and specificity of all included studies, with moderate inter-study heterogeneity. The I^2^ statistics also showed heterogeneity (I^2^ = 72.38 95% CI = 0.00–100.00). First, we conducted a subgroup analysis of the included studies according to three set grouping characteristics: sample size (<50/>50), study design (cross-sectional study/other), and diagnostic reference criteria (VFSS/other). Results the difference of whether the reference criteria were VFSS was statistically significant, indicating that the difference of reference criteria may be the source of heterogeneity among included studies. The results are shown in Table 2.

**Table 2:** Subgroup analysis.

Research characteristics	Number of cases	Sensitivity (95%CI)	*p*-value	Specificity (95%CI)	*p*-value	Global test comparison (*p*-value)
Whether the sample size is greater than 50 cases		0.974(0.798–1.189)	0.804	1.051(0.819–1.349)	0.674	0.848
Yes	5					
No	3					
Whether it was a cross-sectional study		1.113(0.867–0.867)	0.352	1.158(0.887–1.512)	0.202	0.393
Yes	6					
No	2					
The reference standard is VFSS.		1.152(0.97–1.366)	0.079	1.228(1.106–1.364)	**0.004**	**0.011**
Yes	5					
No	3					

Bold values indicates statistically significant.

The bolded parts in the figure represent statistically significant differences (*p* < 0.05).

### 3.5 Sensitivity analysis

**Figure 10:**
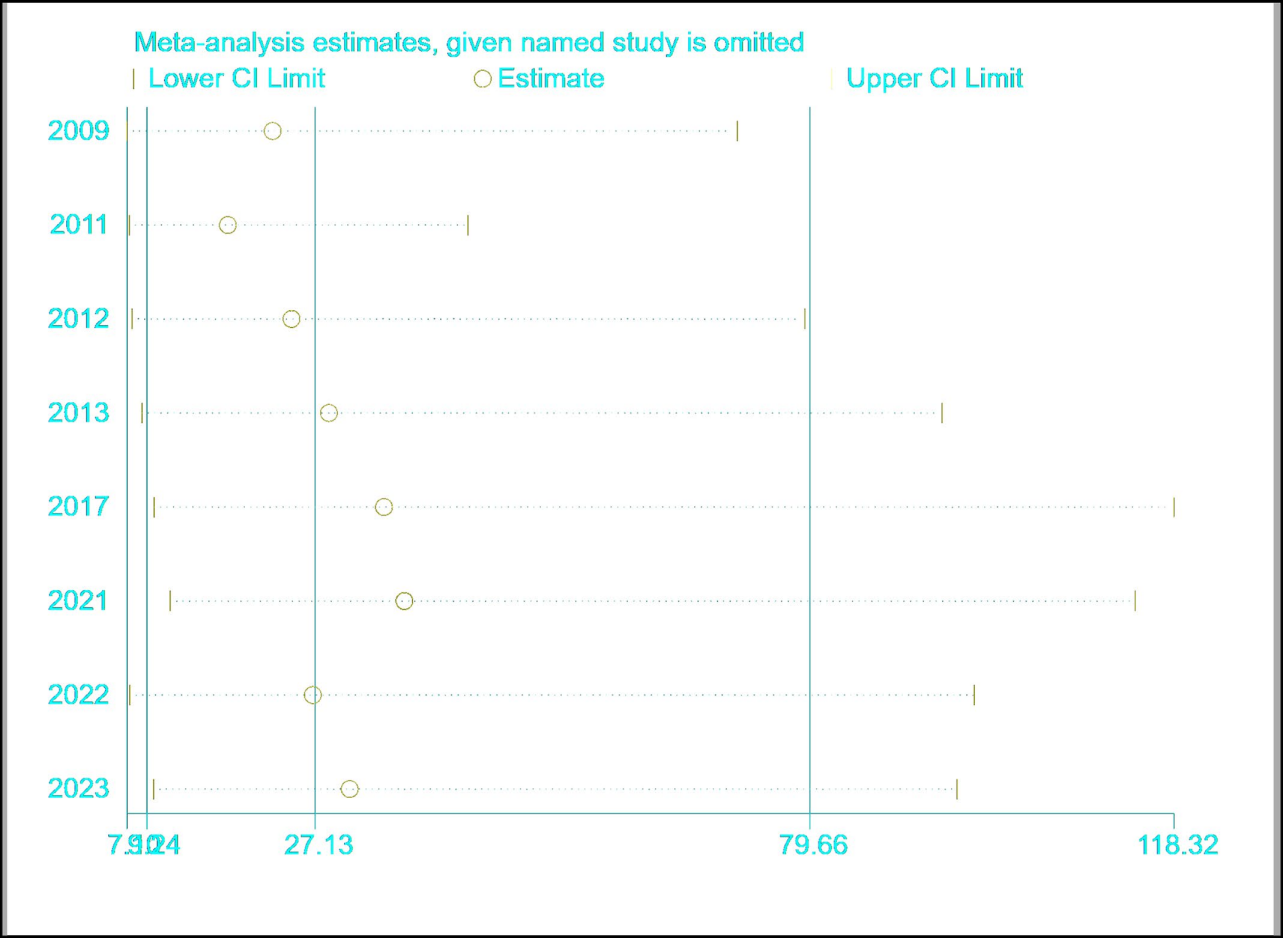
Forest plot of sensitivity analysis for ultrasound-based dysphagia diagnosis.

The results are shown in Figure 10, indicating that the meta-analysis results are relatively stable.

### 3.6 Bias reporting

**Figure 11:**
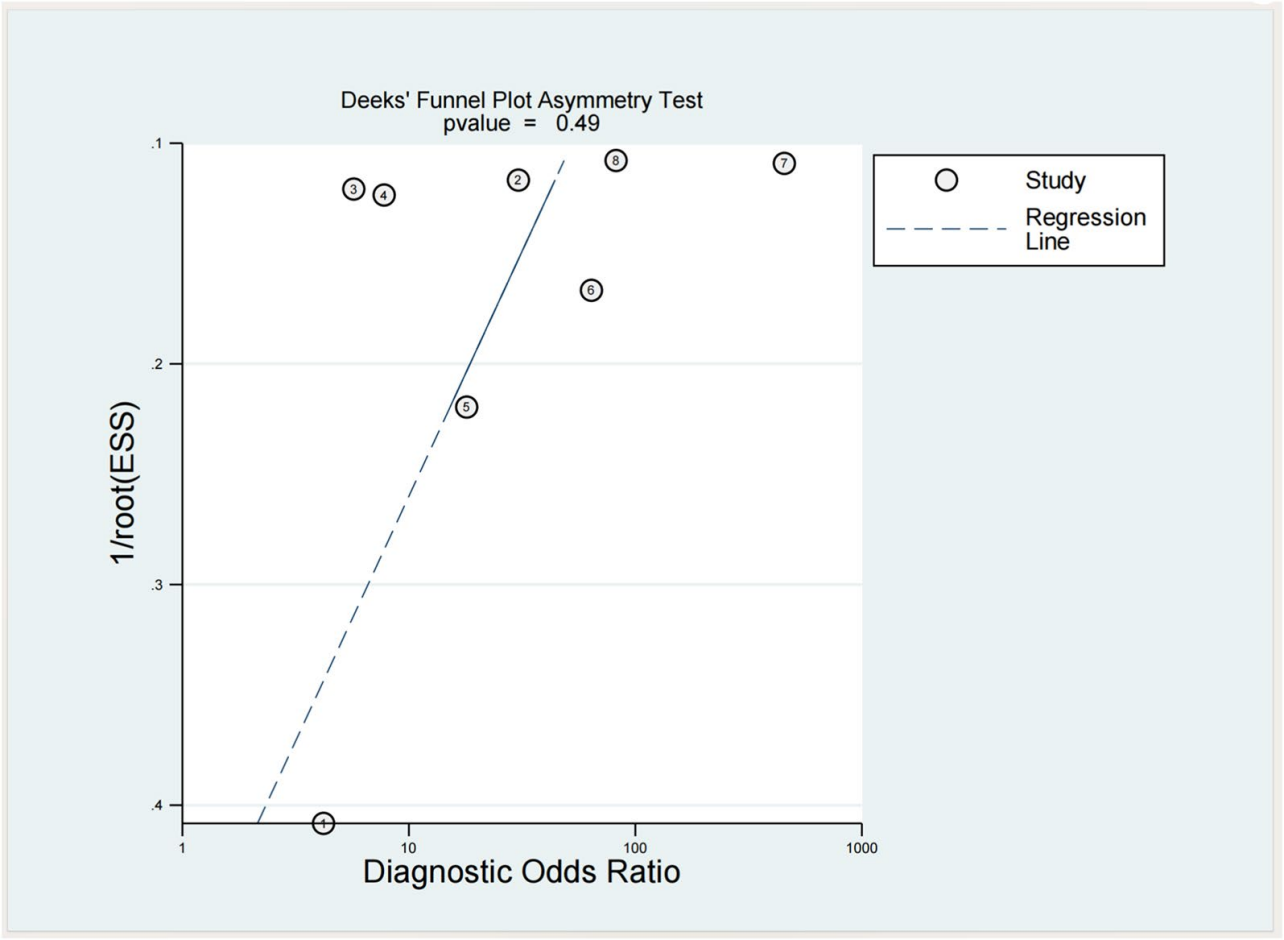
Deeks’ funnel plot asymmetry test for assessing publication bias in ultrasound-related dysphagia studies.

The results are shown in Figure 11, and there is currently no evidence of publication bias based on the *p*-value of Deeks’ Test.

## 4 Discussion

### 4.1 Summary of findings

In this review, the overall sensitivity of ultrasound examination was 0.81 (95% CI 0.73–0.87), and the specificity was 0.86 (95% CI 0.76–0.93), indicating that ultrasound has good diagnostic performance in dysphagia. The results of Bayesian analysis reinforce this finding (Figure 6).

In this study, by quantitatively analyzing the displacement and movement time of the hyoid bone and laryngeal structures during swallowing, we obtained more accurate data (12, 14–17), which provides strong support for the diagnosis of dysphagia. In the study by Deng et al., an advanced image processing algorithm was introduced, using the median flow tracking algorithm to measure hyoid displacement, hyoid movement time, laryngeal displacement, and laryngeal movement time, thereby calculating the hyoid-laryngeal (HL) motion ratio (18). This method avoids the error of manual measurement by automatically tracking the movement trajectory of the hyoid bone and thyroid cartilage in ultrasound images and further improves the precision of data processing by using programming tools such as Python for data analysis. By setting up a control group and conducting reliability assessments, using automated tracking technology of ultrasound image analysis software effectively reduces human measurement errors and enhances diagnostic accuracy.

Most studies used the ROC curve to determine the optimal diagnostic cutoff value and assess diagnostic sensitivity and specificity (13, 15–19). In terms of ultrasound technology, the research team has made optimizations, including improving probe placement techniques, selecting appropriate ultrasound equipment and parameter settings, and standardizing the ultrasound examination process, all of which have improved the sensitivity of ultrasound diagnosis.

Yasuhiro Tomii et al. developed a new ultrasound method (TOFU, i.e., Tongue and Oral Function test with Ultrasound) in their study to assess dysphagia in patients with acute stroke. The TOFU method observes tongue movement during swallowing through ultrasound and uses M-mode images to measure the downward and upward speed of tongue movement (18), as well as changes in the position of the hyoid bone.

Xiao Mingyan et al. applied submental ultrasound examination (SUS) in their study to measure changes in tongue thickness and hyoid bone displacement during swallowing in stroke patients. The study used a self-designed ultrasound machine equipped with a linear array transducer with a frequency of 9 MHz, which may help improve image quality and measurement accuracy (15).

Statistical analysis used multivariate analysis to consider various influencing factors. Through Logistic regression analysis, key factors affecting dysphagia were screened, enhancing the comprehensiveness and accuracy of the diagnosis (12, 16, 18). By assessing inter-rater and intra-rater reliability, the consistency and reliability of the diagnostic results were ensured. Ultimately, by combining clinical assessment and ultrasound examination results, a comprehensive improvement in diagnosis was achieved (15, 17).

This study used meta-analysis to explore the accuracy of ultrasound examination in diagnosing dysphagia and noted moderate heterogeneity among the studies. For a long time, ultrasound has been used to assess the swallowing process, but there have been no conclusive results. Many factors can affect the accuracy of ultrasound diagnosis. To better understand the source of this heterogeneity, we conducted subgroup analysis and meta-regression. The analysis results suggest that different reference standards may significantly affect the accuracy of the study results. Current assessment methods include scale assessment and objective assessment with the aid of equipment. Scale assessment, due to its lack of precision, can be used as a screening or preliminary assessment method (20). Manometry, videofluoroscopic swallowing function examination, and fiberoptic endoscopic swallowing function assessment can objectively assess swallowing function (7, 21, 22).

Videofluoroscopic swallowing function examination is the gold standard for diagnosing dysphagia. However, its radiation exposure is a significant drawback that poses risks to a wide range of patients (7, 23). In contrast, ultrasound, with its portability, non-radiation, and repeat-examination capabilities, is highly recommended in clinical settings. It is not only useful for swallowing assessment but also plays a crucial role in preventing and treating dysphagia (24, 25).

In dysphagia treatment, ultrasound serves as a biofeedback tool during rehabilitation. It shows patients the movement of swallowing-related structures like the tongue, hyoid bone, and laryngeal muscles. This helps patients control muscle movements during exercises, improving the effectiveness of rehabilitation (26).

Ultrasound is also important for monitoring treatment progress. Regular scans can track changes in the structure and function of swallowing-related muscles. For example, it can measure muscle thickness and contractility in cases of muscle atrophy. This data allows doctors to adjust treatment plans (27).

For muscle-related dysphagia, ultrasound-guided injections are a precise option. It enables accurate delivery of medications like botulinum toxin into spastic muscles, reducing side - effects. After injection, ultrasound can monitor the muscle’s response (28).

Moreover, ultrasound can be combined with other treatments. When paired with electrical stimulation therapy, it helps optimize muscle activation. By visualizing muscle contractions, therapists can adjust the electrical stimulation settings for better results (29).

In ultrasound assessment, the choice of examination site is also a key factor. Previous studies have focused on the role of a single structure such as the tongue, geniohyoid muscle, and hyoid bone during the swallowing process (14). In recent years, related research has expanded this perspective, considering the hyoid bone as a highly mobile biomechanical landmark during swallowing, not only as the attachment point for the oral floor, tongue, and laryngeal muscles and non-muscular tissues but also reflecting the relative displacement amplitude of the hyoid bone in the distance changes between the hyoid bone and the mandible (12, 17, 18).

In addition, the accuracy of ultrasound examination may be affected by various factors, including the quality of the equipment used, individual patient differences, and the technical level and interpretation ability of the operator (30, 31).

Although the preliminary results of the likelihood ratio scatter plot indicate that ultrasound examination may have limited utility in diagnosing dysphagia, we believe this may be related to the way the impact of test results on the patient’s probability of illness is evaluated. To obtain a more comprehensive assessment, we adopted the Fagan diagram, which takes into account the changes in both prior and posterior probabilities, providing deeper insights for clinical decision-making. However, the contradiction between the two analysis results suggests that more high-quality studies are needed.

Although there is controversy, we believe that ultrasound examination can serve as a powerful supplement to other diagnostic methods such as videofluoroscopic swallowing examination (VFSS), providing more comprehensive diagnostic information. In particular, some portable ultrasound devices allow for bedside operation, which is particularly beneficial for patients who are unable to move (25).

Due to the comfort and non-invasive nature of ultrasound examination, patients are more likely to cooperate with the examination, especially in cases where multiple assessments are needed. Ultrasound assessment can help doctors understand the specific type and extent of the patient’s dysphagia and formulate personalized treatment plans. By monitoring the recovery process of swallowing function, an ultrasound examination helps to adjust the rehabilitation plan. Due to the safety of ultrasound examination, it can reduce the medical risks that patients may encounter during the diagnostic process. Compared to other diagnostic methods, ultrasound images can serve as educational tools to help patients and their families understand the nature and treatment process of dysphagia. Ultrasound examination is cost-effective, reducing the burden of treatment (32, 33).

Our study included studies related to neurological system diseases, which have a close relationship with dysphagia (34). The act of swallowing is a finely coordinated physiological process that depends on the synergistic work of muscles and nerves in the mouth, pharynx, and esophagus (2). These muscles and nerves are precisely regulated by the nervous system to ensure that food and liquids can safely pass through the digestive tract. When the nervous system is affected by disease, it may lead to damage to swallowing function, thereby causing dysphagia. Notably, stroke and cerebral palsy (CP) exemplify distinct neurological etiologies of dysphagia with contrasting mechanisms: stroke typically induces dysphagia via acute damage to cortical swallowing centers or brainstem nuclei, leading to muscle paresis or reflex impairment, while CP stems from developmental brain injuries causing spasticity, ataxia, or hypotonia in swallowing muscles, often resulting in prolonged oral food manipulation or pharyngeal residue due to laryngeal closure defects. Dysphagia not only affects an individual’s ability to eat but may also be directly related to the occurrence of malnutrition, and the severity of the disease often exacerbates the risk of malnutrition (35).

Currently, the assessment of swallowing function usually depends on some technologies that are somewhat invasive or costly, such as videofluoroscopic swallowing study (VFSS) or fiberoptic endoscopic swallowing assessment (FEES) (25). Although these methods provide detailed information on swallowing function, their application is limited to specific clinical environments and economic conditions.

With the advancement of medical technology, ultrasound examination, as an emerging assessment tool, has begun to show great potential in the assessment of swallowing function due to its non-invasive nature, cost-effectiveness, and portability. Ultrasound technology uses high-frequency sound waves for imaging, which can observe and assess key muscles involved in the swallowing process, such as the geniohyoid muscle, digastric muscle, and mylohyoid muscle, in detail. It can not only display the movement of muscles but also monitor the transmission process of food in the oral and pharyngeal cavity in real - time, which benefits from the advancement of medical technology (25).

Technological innovations, such as the application of wireless probes and flat panel monitoring devices, make ultrasound examination more flexible and easy to operate, further enhancing its practicality in clinical assessment (36, 37). These advantages of ultrasound examination make it a potentially important tool to supplement or replace traditional assessment methods, especially in situations where rapid, bedside assessment is needed.

In summary, the impact of neurological diseases on swallowing function is multifaceted, and ultrasound examination, as an emerging evaluation tool, provides a new perspective for the diagnosis and treatment of dysphagia. In the future, with continuous technological advancements and in-depth clinical application, ultrasound examination is expected to play an increasingly important role in the field of swallowing function assessment.

### 4.2 Assessment of the strengths and limitations of this review

This review has both strengths and limitations that need to be considered when looking at the results. The strengths are that it did a comprehensive within the English and Chinese language literature search for studies, which made it more representative. It used traditional meta-analysis along with Bayesian analysis like Fagan diagrams and likelihood ratio matrices to give good info for clinical use. The limitation is that there is a lot of heterogeneity in the studies. The design, patient population, and diagnostic criteria are all different. Despite having explored factors like different reference standards, study designs, and sample sizes as potential sources of heterogeneity, the exact contribution and relative importance of each factor remain unclear, and there may be other unidentified factors contributing to the observed heterogeneity. In addition, in this study, the included articles were only from China and Japan, and in the process of screening the articles, we found that the number of relevant studies on ultrasonic diagnosis of dysphagia in other regions was small. This phenomenon may be caused by several factors. Different regions have different medical research priorities and resource allocation. Some regions may focus more on other disease areas or diagnostic techniques, and pay less attention to the ultrasonic diagnosis of dysphagia. At the same time, differences in research methods and diagnostic criteria may also make it difficult for studies in some areas to meet the inclusion criteria of this study.

This limitation has an impact on the generality of the findings. The Chinese and Japanese populations have certain similarities in genetic background, living habits, and disease spectrum, but there are many differences between different populations in different regions, which may affect the accuracy and effectiveness of ultrasonic diagnosis of dysphagia. For example, the physiological structure of swallowing in different ethnic groups may be slightly different, and the underlying diseases with high incidence in some areas may also interact with dysphagia, which may affect the effect of ultrasound diagnosis. Therefore, the sensitivity and specificity of ultrasonic diagnosis of dysphagia based on the results of the present study may not be directly applicable to other populations.

In evaluating the results, we must take into account both the advantages and disadvantages. The useful parts of the review are there, but heterogeneity and other issues mean we have to be careful. The true value of ultrasound in different clinical settings for dysphagia needs to be further studied.

### 4.3 Applicability of the findings to the review question

This review applies to healthcare providers who need to assess dysphagia. As expected, heterogeneity is an issue. Although we have considered several factors that could contribute to the heterogeneity, including differences in patient populations, diagnostic criteria, and study designs, the complexity of these relationships means that we cannot fully account for all sources of heterogeneity, and the relative impact of each factor on the overall results is still not well - defined. Therefore, factors that may affect the performance of ultrasound as a diagnostic tool cannot be proposed.

## 5 Conclusion

### 5.1 Implications for practice

Our study results partially support the use of ultrasound for diagnosing dysphagia. Although there is currently insufficient evidence to show that ultrasound can be used as an independent tool for diagnosing dysphagia, the diagnostic performance of ultrasound may have certain clinical application value for diagnosing this disease. Ultrasound examination can serve as a supplement to other diagnostic methods (such as videofluoroscopic swallowing examination), helping to provide more comprehensive diagnostic information. Some devices can also be examined at the bedside, suitable for patients with limited mobility. Given the existing evidence of heterogeneity, caution is needed when interpreting the results. Overall, we need to clarify the heterogeneity and its sources more clearly to draw a clear conclusion, and it is recommended to consider the use of ultrasound examination in routine clinical practice.

### 5.2 Implications for research

Ultrasound examination for dysphagia trials deserves further research. With technological advancements, such as the use of different types of ultrasound devices, B-mode ultrasound diagnostic devices can form a series of brightness-modulated ultrasound cross-sectional images, and M-mode ultrasound diagnostic devices can intuitively display tissue motion in real time. These two types of ultrasound devices are most used in the field of swallowing, and endoscopic ultrasound (EUS) extended from the two has also been applied to the field of swallowing. When considering the diagnostic efficacy of ultrasound, other factors can also be considered, including the operator’s technical level. The visualization site of ultrasound, and the research on the diagnostic potential in combination with different tools, is also very important for the diagnosis of dysphagia. Finally, as the quality of ultrasound examination improves, the conclusions of this study need to be reviewed regularly.

### 5.3 Policy relevance

From the perspective of policy relevance, the results of this study have potential implications for the formulation of healthcare policies. Given the promising application prospects of ultrasound in diagnosing dysphagia, healthcare policies should consider shifting medical resources toward ultrasound diagnostic technology. This includes increasing investment in ultrasound equipment, especially in primary healthcare units, to improve the popularity of ultrasound equipment and enable more patients to access convenient ultrasound examination services. Meanwhile, efforts should be intensified to support training related to ultrasound diagnostic technology, cultivating more professional ultrasound diagnosticians and enhancing the overall level of medical services.

In addition, the results of this study can contribute to the update of relevant diagnostic guidelines. Currently, the diagnosis of dysphagia mainly relies on some invasive or costly examination methods, such as videofluoroscopic swallowing study (VFSS) and fiberoptic endoscopic swallowing function assessment (FEES). With the development and in - depth research of ultrasound diagnostic technology, its importance in diagnosis is becoming more prominent. Therefore, when formulating or updating the diagnostic guidelines for dysphagia, relevant content regarding ultrasound examination should be fully considered. It is necessary to clarify its status and role in the diagnostic process, providing more scientific and comprehensive diagnostic guidance for clinicians. Through these measures, not only can the quality of medical services be improved, but also medical costs can be reduced, enabling more rational allocation of limited medical resources and ultimately enhancing patients’ health and quality of life.
