# Supplementary material for: Diagnostic accuracy of ultrasound for dysphagia in neurological disorders including stroke: a systematic review and meta-analysis
Source: Front Neurol. 2025 Aug 21;16:1534173. doi: 10.3389/fneur.2025.1534173 (PMC12408267; doi:10.3389/fneur.2025.1534173)
Supplement: Supplementary file 1 [file Table_1.doc]

PubMed, Embase, CINAHL, Cochrane Library, Web of Science, China National Knowledge Infrastructure, China Science and Technology Journal Library, Wanfang, China Biomedical CD-ROM full-text Database. The bibliography of the search articles was also screened.

PubMed：((deglutition disorder OR dysphagia) AND (ultrasonography OR ultrason OR ultrasound OR echotomography OR "echo tomography" OR echography OR sonography) AND (screening OR predict OR test OR tests OR detect OR assess OR evaluate) AND (deglutitive OR swallow OR esophago OR pharynx OR oro-pharynx))

WOS

https://webofscience.clarivate.cn/wos/alldb/summary/597ab2fa-e8dc-4d83-843e-c7c059a80a85-e62cacb7/relevance/1

(TS=(deglutition disorder) OR TS=(dysphagia)) NOT (SILOID==("PPRN"))

(TS=(Ultrasonography) OR TS=(ultrason) OR TS=(echotomography) OR TS=(echo tomography) OR TS=(echography) OR TS=(sonography) OR TS=(ultra sound) OR TS=(ultrasound)) NOT (SILOID==("PPRN"))

(TS=(screening) OR TS=(predict) OR TS=(test) OR TS=(tests) OR TS=(detect) OR TS=(assess) OR TS=(evaluate)) NOT (SILOID==("PPRN"))

(TS=(deglutitive ) OR TS=(swallow) OR TS=(esophago) OR TS=(pharynx) OR TS=(oro-pharynx)) NOT (SILOID==("PPRN"))

(#5 AND #4 AND #3 AND #2) NOT (SILOID==("PPRN"))

Embase：'swallowing'/exp OR swallowing OR 'deglutition disorder':ti,ab,kw OR dysphagia:ti,ab,kw

'echography'/exp OR echography OR ultrasound:ti,ab,kw OR ultrasonography:ti,ab,kw OR ultrason:ti,ab,kw OR echotomography:ti,ab,kw OR esophago:ti,ab,kw OR 'echo tomography':ti,ab,kw OR echography:ti,ab,kw OR sonography:ti,ab,kw OR 'ultrasound guided biopsy':ti,ab,kw

'ultrasound'/exp

'screening'/exp OR screening OR predict:ti,ab,kw OR ultrasonography:ti,ab,kw OR test:ti,ab,kw OR tests:ti,ab,kw OR detect:ti,ab,kw OR assess:ti,ab,kw OR evaluate:ti,ab,kw

'diagnosis related group'/exp

CINAHL：

swallowing OR dysphagia OR deglutition disorder OR dysphagia

Ultrasonography OR ultrason OR ultrasound OR echotomography OR echo tomography OR echography OR sonography OR ultra sound

screening OR predict OR test OR tests OR detect OR assess OR evaluate OR ultra sound

(screening OR predict OR test OR tests OR detect OR assess OR evaluate OR ultra sound) AND (S1 AND S2 AND S3)

Cochrane

((("swallowing"[Text Word] OR "dysphagia"[Text Word] OR "deglutition disorder"[Text Word]) OR ("Swallows"[Mesh] AND "Swallows"[Mesh].all))

AND (("ultrasonography"[Text Word] OR "ultrasound"[Text Word] OR "sonography"[Text Word] OR "echography"[Text Word]) OR ("Ultrasonography"[Mesh] AND "Ultrasonography"[Mesh].all))

AND ("screening"[Text Word] OR "predict"[Text Word] OR "test"[Text Word] OR "tests"[Text Word] OR "detect"[Text Word] OR "assess"[Text Word] OR "evaluate"[Text Word]))

Chinese database retrieval

China National Knowledge Infrastructure= (Dysphagia + dysphagia) (ultrasound + ultrasound) (Diagnosis + evaluation)

Wan Fang: Topics: (dysphagia OR dysphagia) AND (ultrasound OR ultrasound) AND (Diagnosis OR evaluation)

Pulse: (Title OR keyword = dysphagia OR title OR keyword = dysphagia) AND (title or keyword = ultrasound or title or keyword = ultrasound) AND (title or keyword = diagnosis or title or keyword = evaluation)
